# The interactive functional biases of manual, language and attention systems

**DOI:** 10.1186/s41235-022-00365-x

**Published:** 2022-03-02

**Authors:** Deborah J. Serrien, Louise O’Regan

**Affiliations:** School of Psychology, University of Nottingham, Nottingham, UK

**Keywords:** Laterality, Handedness, Hand dexterity, Word processing, Symmetry detection

## Abstract

Hemispheric lateralisation is a fundamental principle of functional brain organisation. We studied two core cognitive functions—language and visuospatial attention—that typically lateralise in opposite cerebral hemispheres. In this work, we tested both left- and right-handed participants on lexical decision-making as well as on symmetry detection by means of a visual half-field paradigm with various target–distractor combinations simultaneously presented to opposite visual fields. Laterality indexes were analysed using a behavioural metrics in single individuals as well as between individuals. We observed that lateralisation of language and visuospatial attention as well as their relationship generally followed a left–right profile, albeit with differences as a function of handedness and target–distractor combination. In particular, right-handed individuals tended towards a typical pattern whereas left-handed individuals demonstrated increased individual variation and atypical organisation. That the atypical variants varied as a function of target–distractor combination and thus interhemispheric communication underlines its dynamic role in characterising lateralisation properties. The data further revealed distinctive relationships between right-handedness and left-hemispheric dominance for language together with right-hemispheric dominance for visuospatial processing. Overall, these findings illustrate the role of broader mechanisms in supporting hemispheric lateralisation of cognition and behaviour, relying on common principles but controlled by internal and external factors.

**Supplementary Information:**

The online version contains supplementary material available at 10.1186/s41235-022-00365-x.

## Significance statement

Cognitive functions are often asymmetrically organised across the brain, with some dominant in the left hemisphere and others in the right hemisphere. Although the left–right dichotomy is considered to reflect a biological constraint, there are little insights into the factors that drive its variation. One such important trait that has its roots in the brain concerns handedness, which has a 90–10% ratio of right- versus left-handers in the general population. However, research that studies cognitive functions usually only include right-handed participants, which affects interpretation and generalisation of findings. Here, we assessed flexibility of hemispheric processing in both handedness groups for two cognitive functions that are part of diverse aspects of our daily life activities, i.e., language and visuospatial attention. The data revealed that left-handedness enhances individual heterogeneity and deviation from typical organisation of cognitive functioning. Furthermore, the environmental context played a steering role in defining the lateralisation properties. The findings also showed associations between right-handedness and the language and attention systems, indicating that dedicated processing mechanisms assist the perceptual-motor preferences of people within their environment. It suggests that individuals with different types of handedness are distinctively prone to stimuli from the surroundings, influencing how they perceive and respond in everyday, educational and sports settings. These biases in information processing shape immediate choices and judgements, and accordingly how one behaves and learns in external situations such as navigating through and interacting with the changing demands of the environment.

## Introduction

Functional lateralisation is a fundamental principle of brain organisation and refers to differences of the left and right cerebral hemispheres as obtained from brain activation or behavioural performance (Corballis, 1989). It has been proposed that lateralisation evolved in two steps as an evolutionarily stable strategy: individual-level lateralisation occurs when single individuals display a strong preference whereas population-level lateralisation happens when the majority of individuals demonstrate the same asymmetry such that the population is biased. Lateralisation occurred first at the individual-level due to advantages of brain efficiency followed by the population-level because of social coordination as individuals aligned their behaviour with one another (Ghirlanda & Vallortigara, 2004).

Noteworthy is that hemispheric lateralisation can be supported by intrahemispheric and interhemispheric mechanisms. Whereas intrahemispheric connections maintain regional activation within one hemisphere, interhemispheric connections support the communication across hemispheres (Karolis et al., 2019; Tzourio-Mazoyer, 2016). In this respect, the corpus callosum plays a prime role in steering dynamic interactions between both sides, especially between homotopic areas. As cognitive functions often rely on information from both hemispheres, it implies that independent processing can only be advantageous if both sides engage in efficient exchanges with interhemispheric communication mediating the balance. Moreover, it has been proposed that callosal pathways involve influences of cooperation and competition, with excitatory and inhibitory connections that differ across regions (Bloom & Hynd, 2005; Clarke & Zaidel, 1994).

Principles of lateralisation are considered to have contributed to a left-sided population bias of human language, providing a reference for other systems such as visuospatial functions (Kosslyn, 1987; Liu et al., 2009; Price, 2000). Although performance differences between cognitive tasks have been explained by way of distinct processing capacities of the left versus right hemisphere, there is also evidence for differences in attention allocation to the left and to the right (Corbetta & Shulman, 2011; Kinsbourne, 1987; Thiebaut de Schotten et al., 2011). According to Hugdahl (2000), the left–right dichotomy evolved from evolutionary pressures towards an increasing demand for specialisation of key behaviours, i.e., symbolic characterisation of the environment and communication with others versus orientation in unfamiliar environments and identification of objects and their relations. However, there are limited insights into the relationship between lateralised cognitive functions and questions remain as to whether a function lateralised in one hemisphere associates with another function in the opposite hemisphere. This phenomenon, labelled as complementary lateralisation, has been addressed according to two main hypotheses; statistical and causal. Whereas the statistical hypothesis claims that the influences that guide complementary organisation are independent of one another (Groen et al., 2012), the causal hypothesis suggests that lateralisation of a function to one particular side forces the other function to the opposite side (Cai et al., 2013).

Although language and visuospatial attention typically show left–right lateralisation, differences of representation are possible such as crowding (both functions lateralise in left or right hemisphere only) or mirror-reversal (language lateralises in right and visuospatial in left hemisphere), (Cai et al., 2013; Gerrits et al., 2020). Thus, by examining influences that associate with lateralisation patterns, we can gain important insights into the extent of atypical variants. One such factor refers to handedness, which expresses asymmetry of movement control and the underlying mechanisms of the sensorimotor system (Martin et al., 2011; Pool et al., 2014; Serrien & Sovijärvi-Spapé, 2016). Throughout history, a prevalence of right-handedness has been observed in humans at the population-level. More specifically, there is a 90–10% ratio of right- versus left-handers (Coren & Porac, 1977). Both groups show distinct functionality of language and albeit less investigated visuospatial circuits (Flöel et al., 2005; Knecht et al., 2000; Powell et al., 2012; Tussis et al., 2016; Tzourio et al., 1998) in addition to different callosal characteristics (Cherbuin & Brinkman, 2006). Therefore, an experimental approach that examines these lateralised functions in the same individuals is key to unravel mutual influences and to understand the underlying processes.

The aim of this study is to assess the principles of lateralisation and complementary asymmetry of language and visuospatial attention in different environmental contexts and as a function of handedness. This will enable us to evaluate the prevalence of typical organisation and any modulations due to internal and external factors. We use the visual half-field technique to establish indirectly the involvement of the dominant and nondominant hemisphere as well as role of interhemispheric communication (Hunter & Brysbaert, 2008; Perrone-Bertolotti et al., 2013). To this end, the experimental design includes a simultaneous presentation of target and distractor stimuli to opposite visual fields, based on the argument that the presence of the distractor disrupts the interhemispheric interactions and competes with processing of the target. By varying the target–distractor relationship, we can thus assess the role of interhemispheric pathways in supporting lateralisation patterns (Boles, 1990). The hypothesis is that lateralisation and complementarity of function relies on the type and combination of target and distractor stimuli. We further test left- and right-handers on both tasks and hypothesise that handedness affects how the hemispheres weigh their processing demands and interactions with one another. In order to cover the multidimensional nature of handedness, we use preference (self-report) and performance (dexterity) measurements. Therefore, assessing the relationship between hand preference, hand performance and hemispheric asymmetries of cognitive functions is important to strengthen our insights into the mechanisms that underlie functional asymmetries of cognitive functions.

## Material and methods

### Participants

There were 52 participants (*M*_AGE_: 21.8 ± 0.9) with no history of neurological or psychological illness as assessed by a standardised questionnaire. We invited individuals who self-identified as right-handed and non-right-handed.

### Handedness tests

Handedness can be assessed through preference and performance measurements. Whereas hand preference identifies the preferred hand for manual activities and is evaluated via self-report inventories, hand performance contrasts the functional ability of the left and right hand and can be measured by means of behavioural outputs from manual tasks (Bryden, 1982; Corey et al., 2001). Often a stronger performance is observed with the preferred hand, although this is not always the case (Jäncke et al., 1998).

#### Hand preference

Participants completed a handedness questionnaire for manipulation tasks, consisting of 15 items (i.e., write, hold toothbrush, throw ball, hold racquet, use spoon to stir, hold hammer, use scissors, use stapler, open lid from drinks can, use remote control, peel apple, use comb, flip a coin, hold knife to cut, use computer mouse).

#### Hand performance (dexterity)

Participants performed a manual proficiency test using a grooved pegboard. Participants inserted as many pegs as possible using either the left or right hand on alternating trials. Each trial lasted 30 s and there were three trials per hand.

### Experimental tasks: language and visuospatial processing

We used a visual half-field paradigm with three target–distractor combinations: incongruent, congruent, and perceptual. It is argued that the incongruent and congruent distractors interact with the targets at relevant higher-order processing stages, whereas perceptual distractors place a demand on early stages of input such as stimulus perception and identification. All participants completed both the language and visuospatial task in a randomised order, with a break in-between tasks. The experimental designs were created using PsychoPy (Peirce & MacAskill, 2018).

#### Language task

##### Aim

Language comprehension and conceptual processing of word forms are strongly driven by the left hemisphere (Price, 2000). Previously, lexical decision-making tasks with the implementation of words versus non-words have been used to study differences of hemispheric processing for language. Overall, studies have shown an advantage for words when presented in the right visual field and thus with processing in the left hemisphere, but not for non-words (Measso & Zaidel, 1990; Mohr et al., 1994). Here, we use action words that associate with hand activities (e.g., grasp) based on the argument that their meaning is learned through interactions with the action, object or process that are conceptually represented by the words, providing a left-hemispheric bias (Hauk et al., 2004; O’Regan & Serrien, 2018).

##### Procedure

**Fig. 1:**
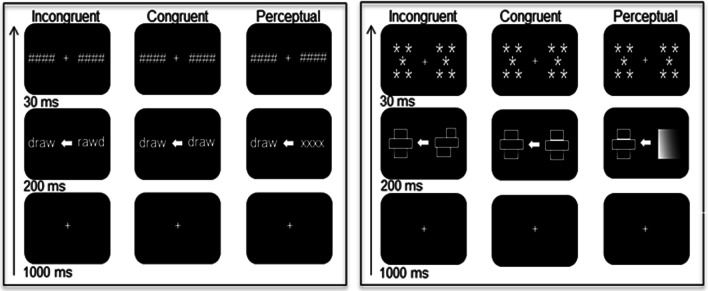
Schematic timeline for the language (left panel) and visuospatial (right panel) task, showing the incongruent, congruent and perceptual conditions

Participants were seated at a viewing distance of 70 cm from a computer monitor, with their head rested on a chinrest. Each trial started with a centrally-presented fixation cross for 1000 ms (Fig. 1, left panel), followed by an arrow pointing either to the left or to the right side for 200 ms alongside a target and distractor. The stimuli consisted of action words, i.e., verbs related to hand use, and non-words. Both target and distractor were matched for length (i.e. 4–5 letters), presented in opposite visual fields and appearing ± 3° of visual angle from the centre of the screen. The target and distractor were replaced by backward masks that matched the stimuli for length and remained on screen for 30 ms. Each trial was followed by a 1000 ms inter-stimulus interval. All stimuli were presented in white Arial font on a black background, and subtended 1.1° of visual angle in height.

The target–distractor combination included three conditions; (1) the target and distractor consisted of different lexical categories (incongruent), (2)*,* the target and distractor consisted of two identical words or two identical non-words categories (congruent), (3) the word or non-word target was presented alongside a meaningless letter string (perceptual). There were 20 action words and 20 non-words, which resulted in 240 trials with two blocks for congruent and incongruent conditions and one block for perceptual condition. There were 40 observations per participant and per condition. Trials were randomised within blocks of trials. Participants were instructed to indicate whether the target was a word or non-word by means of a bimanual response, i.e. pressing two keys simultaneously with both index or middle fingers*.* They received practice trials with feedback and were offered breaks between blocks of trials.

#### Visuospatial task

##### Aim

Visuospatial processing is often lateralised to the right hemisphere (Vogel et al., 2003), and can be captured by a left visual field advantage such as for spatial location (Wilkinson & Halligan, 2002). This bias is proposed to draw upon right-hemispheric dominance for spatial attention, which sets priorities to selectively process information in space (Corbetta & Shulman, 2011; Petersen & Posner, 2012). Here, we study symmetry detection as a representative visuospatial function that refers to the identification of the property of objects whereby the parts are related with one another through an imaginary axis (Verma et al., 2013; Wagemans, 1995). It occurs in many configurations but we focus on the condition where the central axis is vertical*.* Previous work has revealed that symmetry detection is preferentially identified in the left visual field and thus with processing in the right hemisphere (Verma et al., 2013).

##### Procedure

The procedure was similar as for the language task. The target and distractor stimuli were two-dimensional figures created in white lines against a black background, and were up to 4° wide and 2° high. They consisted of three horizontal rectangles that were placed on top of each other, resulting in symmetrical or asymmetrical images along the vertical axis (Fig. 1, right panel).

The target–distractor combination involved three conditions: (1), the target and distractor consisted of images from different spatial categories (incongruent), (2)*,* the target and distractor consisted of two identical symmetrical or two identical asymmetrical categories (congruent), (3) the symmetric or asymmetric target was presented alongside a greyscale image (perceptual). Stimuli consisted of 20 symmetrical and 20 asymmetrical images, resulting in 240 trials with two blocks for congruent and incongruent conditions and one block for perceptual condition. There were 40 observations per participant and per condition. Trials were randomised within blocks of trials. Participants were instructed to indicate whether the target was a symmetrical or asymmetrical image by means of a bimanual response. They received practice trials with feedback and were offered breaks between blocks of trials.

### Measurements

#### Measurements handedness tests

The handedness questionnaire used a 5-point Likert scale that ranged between always left (0), usually left (1), equal (2), usually right (3), and always right (4). For each participant, the scores of the items were added, divided by the maximum score of the questionnaire, and multiplied by 100. This provided a handedness score that varied between 0 (extreme left-handedness) and 100 (extreme right-handedness). The handedness scores were used to categorise the participants into 25 left-handers (*M*_HAND_: 24.5 ± 2.7; *M*_AGE_: 23.8 ± 1.7**,** 18 females) and 27 right-handers (*M*_HAND_: 90.0 ± 1.5; *M*_AGE_: 20.0 ± 0.6, 24 females), with values < 50 indicating a left-hander and values > 50 specifying a right-hander. The writing hand was also a condition as most people define their handedness through their writing hand. Performance on the pegboard test was measured for each hand by obtaining an average score from the trials. We calculated a laterality index (LI_PEG_) to quantify the between-hand asymmetry using the formula [*R* − *L*]/[*R* + *L*] × 100, where *R* and *L* represented the right and left hand performance. Positive scores represented a right hand advantage.

#### Measurements visual half-field tasks

Performance on the cognitive tasks was measured using reaction time (i.e., averaged bimanual responses) and accuracy (i.e., percentage of correct responses). We calculated a laterality index to express hemispheric asymmetry, for reaction time (LI_TIM_) according to [*L* − *R*]/[*L* + *R*] × 100 and for accuracy (LI_ACC_) according to [*R* − *L*]/[*R* + *L*] × 100 where *R* and *L* stimuli were presented in the right and left visual field. Positive scores of LI_TIM_ and LI_ACC_ referred to a right visual field advantage (left hemisphere), whereas negative scores of LI_TIM_ and LI_ACC_ represented a left visual field advantage (right hemisphere). Various methods of classification and cut-off scores exist (Michel, 2021). Here, a cut-off score of 0.1 was used to categorise individuals with LI values referring to right visual field (LI >  + 0.1), left visual field (LI <  − 0.1) and non-lateralised (− 0.1 ≤ LI ≤  + 0.1) dominance. The latter third group was included to match the LI_PEG_ data as some participants showed no between-hand performance difference for the pegboard task.

### Statistical analyses

#### Dexterity

The performance scores were analysed using mixed 2 × 2 ANOVA. There was a within-subjects factor of Hand (left vs. right) and a between-subjects factor of Handedness Group (left-handers vs. right-handers). The LI_PEG_ was analysed using independent *t*-test on Handedness Group.

#### Language and visuospatial task

Reaction times and accuracy scores were analysed using mixed 2 × 2 × 3 × 2 ANOVAs. There were three within-subjects factors of Visual Field (left vs. right), Target (word vs. non-word for language, or, symmetric vs. asymmetric for visuospatial) and Distractor (incongruent vs. congruent vs. perceptual) and a between-subjects factor of Handedness Group. The LI_TIM_ and LI_ACC_ were analysed for word targets (language) and symmetric targets (visuospatial) using mixed 3 × 2 ANOVAs, including a within-subjects factor of Distractor and a between-subjects factor of Handedness Group. A focus was on the word and symmetric targets as these have the strongest intrinsic significance that across both domains.

#### Individual- and population-level lateralisation

Most studies focus on population-level lateralisation but individuals often show their own preference, pointing to individual variation. Here, we assess both types of organisation. *First*, individual-level lateralisation implies that each individual has their own preference towards one side. Therefore, we examined the number of individuals who preferred one side (either left or right) as compared to those who did not show a preference (non-lateralised). *Second*, population-level lateralisation indicates that the majority of individuals within a population share the same bias. Thus, we investigated the number of left- and right-handed individuals who showed a similar preference.

#### Individual- and population-level complementary lateralisation

Most studies assess one cognitive task in individuals, but this limits insights into relationships between functions and existence of typical versus atypical complementarity. Here, we examine these types of organisation for each distractor condition separately. *First*, individual-level complementarity suggests that each individual has their own bias. Therefore, we assessed the number of individuals who demonstrated either a typical or atypical profile as well as those who lacked a clear pattern (non-lateralised). *Second*, population-level complementarity suggests that the majority of individuals within a population has the same preference. Hence, we evaluated the number of left- and right-handed individuals who demonstrated a similar bias.

#### Correlation and frequency analyses

Associations were determined using correlations between: (1) handedness tests, (2) handedness tests and language/visuospatial tasks, (3) language and visuospatial tasks for assessing their complementarity. Whereas the statistical hypothesis predicts no correlation between both functions due to independent influences (Groen et al., 2012), the causal hypothesis suggests a negative correlation as a result of dependent influences (Cai et al., 2013). In addition, comparisons were evaluated by means of (1) chi-square tests of the frequency counts, (2) one-sample proportion tests against the null hypothesis of the percentage counts.

#### Results section

We present the reaction time data as the timing measurement is most sensitive to these effects of processing. The accuracy data can be found in the Additional file 1. Mean ± SE are reported.

## Results

### Hand dexterity

#### Pegboard

Both groups performed best with their preferred hand. That is, the analysis revealed a significant interaction between Handedness Group and Hand, *F*(1,50) = 59.99, *p* < 0.001, *η*^2^*p* = 0.55, with opposite performances for left-handers, *t*(24) = 2.60, *p* = 0.016 (left hand: 12.5 ± 0.3; right hand: 11.9 ± 0.3), and right-handers, *t*(26) =  − 8.45, *p* < 0.001 (left hand: 10.9 ± 0.3; right hand: 12.8 ± 0.3). Both groups further differed for their left hand, *t*(50) = 3.63, *p* < 0.001, and right hand, *t*(50) =  − 2.09, *p* = 0.041*. A* significant main effect of Hand was also noted, *F*(1,50) = 16.07, *p* < 0.001, *η*^2^*p* = 0.24.

#### LI_PEG_ (dexterity)

Both groups showed a lead of their dominant hand during task performance. Namely, the analysis demonstrated a significant performance difference between both groups, *t*(50) =  − 7.89, *p* < 0.0001, revealing a left hand lead for left-handers (− 2.4 ± 0.9) versus right hand lead for right-handers (7.9 ± 0.9).

#### LI_PEG_ (dexterity) and handedness scores

**Fig. 2:**
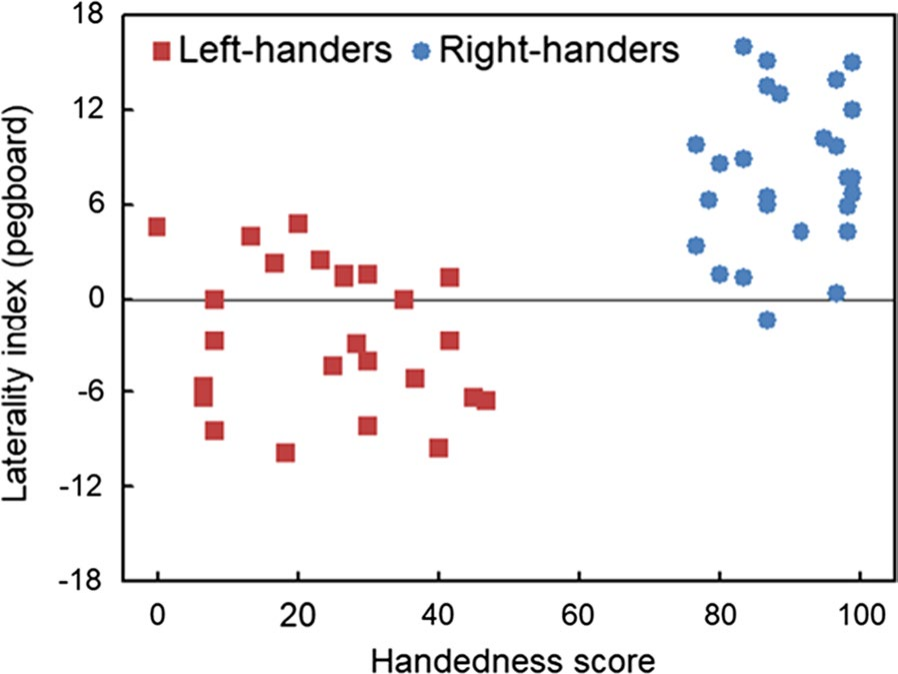
Scatter plot of the laterality index (pegboard) alongside the handedness scores. Pegboard performances with positive versus negative score represent right versus left hand advantage, left-handed < 50 and right-handed > 50

Stronger right-handedness associated with an increased right hand advantage for the pegboard task, which was expressed by a correlation analysis, *r*(50) = 0.69, *p* < 0.001 (Fig. 2). Of note is that a number of left-handers achieved a positive score (*N* = 9, 36%) as compared to right-handers who obtained a negative score (*N* = 1, 4%). Two left-handers showed no between-hand performance difference.

### Language task

#### Mean reaction time

Responses to word targets were faster in the right than left visual field whereas those to non-word targets were unaffected by visual field of presentation. In addition, responses to word targets were quicker than to non-word targets, except in the left visual field alongside a non-word distractor. These effects were expressed as a Visual field × Target × Distractor interaction, *F*(2,100) = 7.04, *p* = 0.001, *η*^2^*p* = 0.13, revealing that word target responses were fastest in the right visual field (*p* < 0.001) whereas no effect was noted for non-word targets. Furthermore, word target responses were quicker than to non-word targets (*p* < 0.001), except in the left visual field with incongruent distractors**.** There were also significant two-way interactions of Visual Field × Target, *F*(1,50) = 11.80, *p* = 0.001, *η*^2^*p* = 0.19, and of Target × Distractor, *F*(2,100) = 7.75, *p* < 0.001, *η*^2^*p* = 0.14, alongside significant main effects of Visual Field, (1,50) = 5.23, *p* = 0.026, *η*^2^*p* = 0.09, Target, *F*(1,50) = 40.97, *p* < 0.001, *η*^2^*p* = 0.45, and Distractor, F(2,100) = 17.60, *p* < 0.001, *η*^2^*p* = 0.26. The reaction times in the left and right visual field for non-words were 818 ± 18 ms and 839 ± 19 ms (incongruent); 836 ± 23 ms and 832 ± 18 ms (congruent); 799 ± 19 ms and 784 ± 20 ms (perceptual), and for words these were 842 ± 22 ms and 770 ± 19 ms (incongruent); 799 ± 21 ms and 759 ± 21 ms (congruent); 733 ± 18 ms and 704 ± 16 ms (perceptual).

#### LI_TIM_ (word targets)

Right visual field dominance varied as a function of distractor type with the strongest asymmetry when there was a non-word distractor. That is, the analysis showed a significant main effect of Distractor, *F*(1,50) = 3.40, *p* = 0.04, *η*^2^*p* = 0.06, with the largest effect for the incongruent (4.4 ± 1.1) followed by congruent (2.6 ± 1.2) and perceptual (1.9 ± 0.6) condition, with the former and latter differing from one another, *p* = 0.03.

##### LI_TIM_ (word targets) and handedness scores

**Fig. 3:**
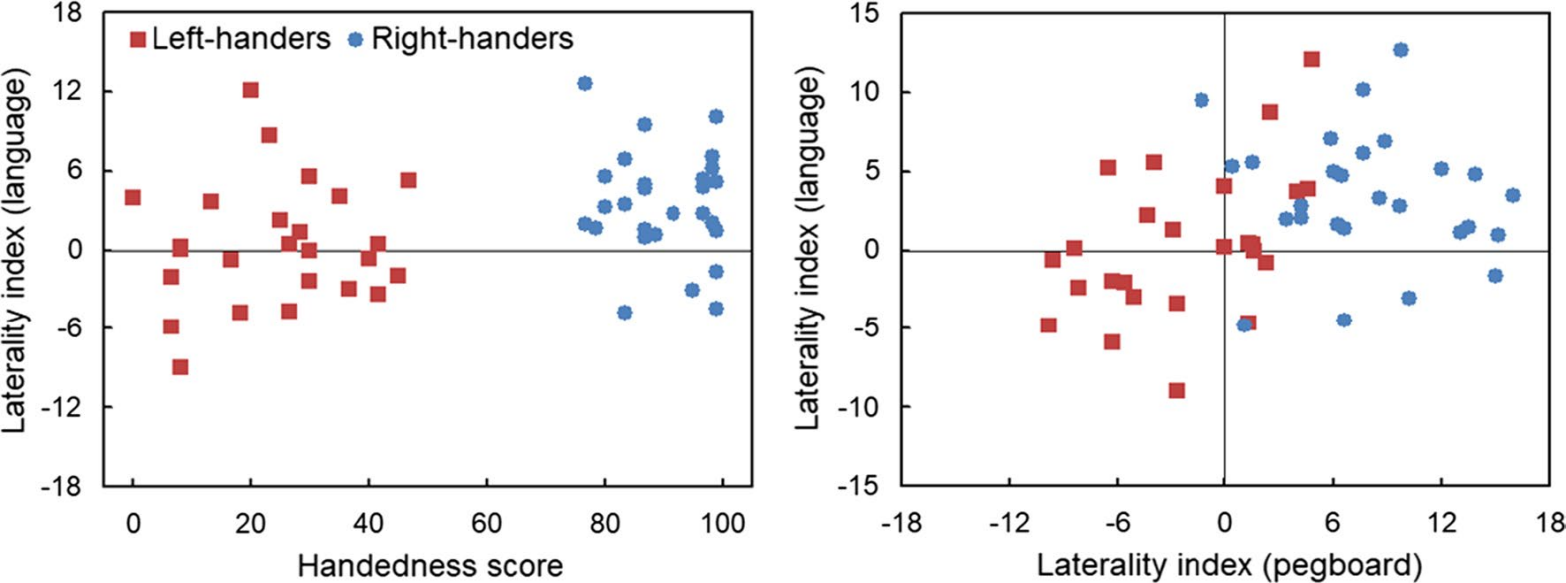
Scatter plots of the laterality index of word targets with handedness scores (left panel) and with the laterality index of pegboard task (right panel). Language responses with positive score represent dominance of right visual field, left-handed < 50 and right-handed > 50, and pegboard performance with positive score indicate right hand advantage

Stronger right-handedness associated with superiority of the right visual field for the perceptual condition only. This was captured by a correlation analysis, *r*(50) = 0.32, *p* = 0.02 (Fig. 3, left panel).

##### Individual- and population-level analysis

*First*, at the individual-level, right-handers provided superior responses in the right visual field whereas this was not the case for left-handers. That is, although lateralisation (left or right) prevailed as opposed to non-lateralisation (*N* = 1), there were differences between left- and right-handed individuals. In particular, more right-handers demonstrated dominant processing in the right visual field (*N* = 23, 85%) than left visual field (*N* = 4, 15%), *χ*^2^ = 47.61, *p* = 0.0001, whereas left-handers showed equal numbers for both visual fields (left: *N* = 13, 52%, right: *N* = 11, 44%), *p* > 0.05. *Second*, at the population-level, the majority of individuals showed dominance of the right visual field. Moreover, there was prevalence of the right visual field (*N* = 34, 65%) against the null-hypothesis with 33.3% according to three categories, *z* = 4.85, *p* < 0.0001**,** 95% CI: 50.52–77.70.

#### LI_TIM_ (word targets) and LI_PEG_ (dexterity)

A stronger right hand advantage associated with superiority of the right visual field for the perceptual condition only. This was expressed by a correlation analysis, *r*(50) = 0.35, *p* = 0.01 (Fig. 3, right panel).

##### Individual- and population-level analysis

*First,* at the individual-level, performers with a right hand advantage showed dominance of the right visual field whereas those with a left hand advantage demonstrated no superiority. That is, although lateralisation (left or right) dominated as opposed to non-lateralisation (*N* = 1), there were differences between performers with a right versus left hand advantage. In particular, more performers with a right hand advantage had dominance of the right visual field (*N* = 28, 80%) than left visual field (*N* = 6, 17%), *χ*^2^ = 39.63, *p* = 0.0001. Conversely, performers with a left hand advantage showed similar dominance of the left visual field (*N* = 9, 60%) and right visual field (*N* = 6, 40%), *p* > 0.05. In addition, there were two participants with no between-hand pegboard performance who obtained right visual field dominance. *Second,* at the population-level (including those with hand advantage), the majority of the performers demonstrated dominance of the right visual field. In particular, there was prevalence of the right visual field (*N* = 34, 68%) against the null-hypothesis with 33.3% according to three categories, *z* = 5.21, *p* < 0.0001, 95% CI: 53.30–80.48.

### Visuospatial task

#### Mean reaction time

Right-handers responded quicker to targets in the left than right visual field whereas left-handers showed no preference. Furthermore, responses to left as compared to right visual field targets were faster but only with non-perceptual distractors. In addition, responses to symmetric versus asymmetric targets were quicker but only with identical distractors. These effects were expressed as a significant Handedness Group × Visual Field interaction, *F*(1,50) = 5.13, *p* = 0.027, *η*^2^*p* = 0.09, demonstrating that right-handers had faster responses to targets in the left than right visual field (753 ± 18 ms vs. 774 ± 19 ms) whereas there were no differences across visual fields for left-handers (750 ± 16 ms vs. 745 ± 20 ms). There was also a significant Visual Field × Distractor interaction, *F*(2,100) = 3.10, *p* = 0.04, *η*^2^*p* = 0.06**,** revealing faster responses to left versus right visual field targets for incongruent (768 ± 13 ms vs. 793 ± 14 ms, *p* = 0.005) and congruent (753 ± 14 ms vs. 774 ± 15 ms, *p* = 0.02) but not for perceptual (722 ± 17 ms vs. 725 ± 16 ms) condition. There was further a significant Target × Distractor interaction, *F*(2, 100) = 18.20, *p* < 0.001, *η*^2^*p* = 0.27, denoting that responding to symmetric versus asymmetric targets was quicker for congruent condition (744 ± 16 ms vs. 789 ± 14 ms (*p* < 0.001) with no difference in the other conditions (incongruent: 783 ± 15 ms vs. 771 ± 14 ms; perceptual: 714 ± 15 ms vs. 734 ± 18 ms). There were also significant main effects of Visual Field, *F*(1,50) = 7.80, *p* = 0.007, *η*^2^*p* = 0.13, and Distractor, *F*(2,100) = 12.12, *p* < 0.001, *η*^2^*p* = 0.19.

#### LI_TIM_ (symmetric targets)

Left visual field dominance varied as a function of handedness and distractor type, revealing superiority for right- but not for left-handers, and the strongest advantage when there was an asymmetrical distractor. That is, the analysis demonstrated a significant main effect of Handedness Group, *F*(1,50) = 4.34, *p* = 0.04, *η*^2^*p* = 0.08, indicating that right-handers had left visual field superiority (− 2.1 ± 0.6) as compared to left-handers (0.0 ± 0.8). There was also a significant main effect of Distractor, *F*(2, 100) = 3.38, *p* < 0.04, *η*^2^*p* = 0.06, illustrating strongest dominance for the incongruent (− 1.9 ± 0.8) followed by congruent (− 1.2 ± 0.6) and perceptual (− 0.1 ± 0.6) condition, with the former and latter differing from one another (*p* = 0.03).

#### LI_TIM_ (symmetric targets) and handedness scores

**Fig. 4:**
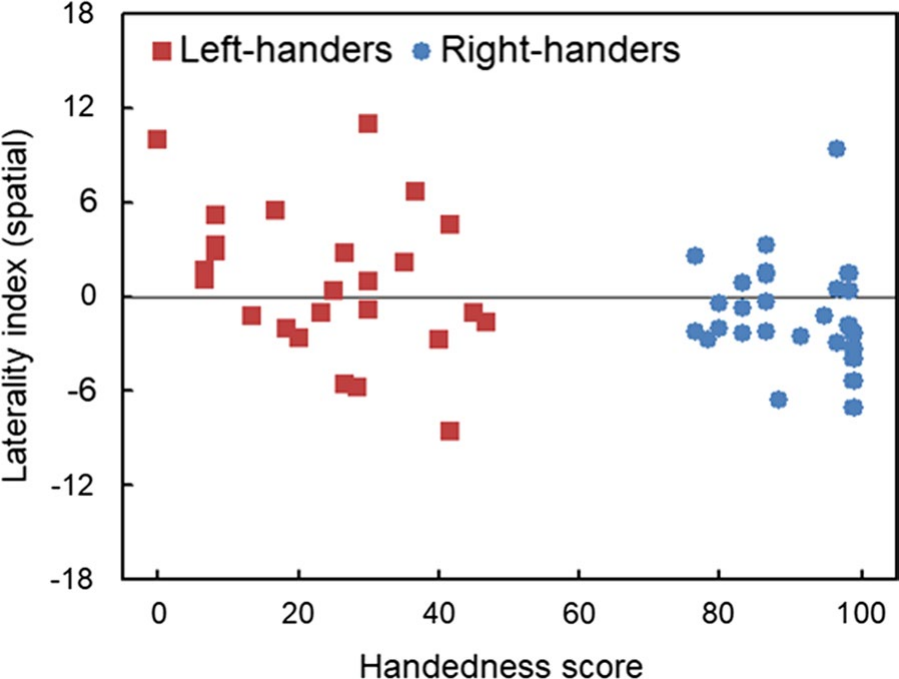
Scatter plot of the laterality index of symmetric targets alongside the handedness scores. Visuospatial responses with negative score represent superiority of the left visual field, left-handed < 50 and right-handed > 50

Stronger right-handedness associated with enhanced dominance of the left visual field for perceptual distractors only. This was supported by a correlation analysis, *r*(50) =  − 0.33, *p* < 0.02 (Fig. 4).

##### Individual- and population-level analysis

*First,* at the individual-level, right-handers provided dominant responses in the left visual field whereas this was not the case for left-handers. That is, although lateralisation (left or right) prevailed as opposed to non-lateralisation (*N* = 0), there were differences between left- and right-handed individuals. In particular, many right-handers showed dominance of the left visual field (*N* = 18, 67%) as opposed to right visual field (*N* = 9, 33%), *χ*^2^ = 10.89, *p* = 0.001, whereas no differences were noted for left-handers (left: *N* = 14, 56% and right: *N* = 11, 44%), *p* > 0.05. *Second*, at the population-level, the majority of the individuals demonstrated prevalence of the left visual field (*N* = 32, 62%) against the null-hypothesis with 33.3% according to three categories, *z* = 4.39, *p* < 0.0001, 95% CI: 47.48–75.10.

#### LI_TIM_ (symmetric targets) and LI_PEG_ (dexterity)

Correlation analysis revealed no significant effect, *p* > 0.05.

### Language and visuospatial tasks: complementary lateralisation

**Fig. 5:**
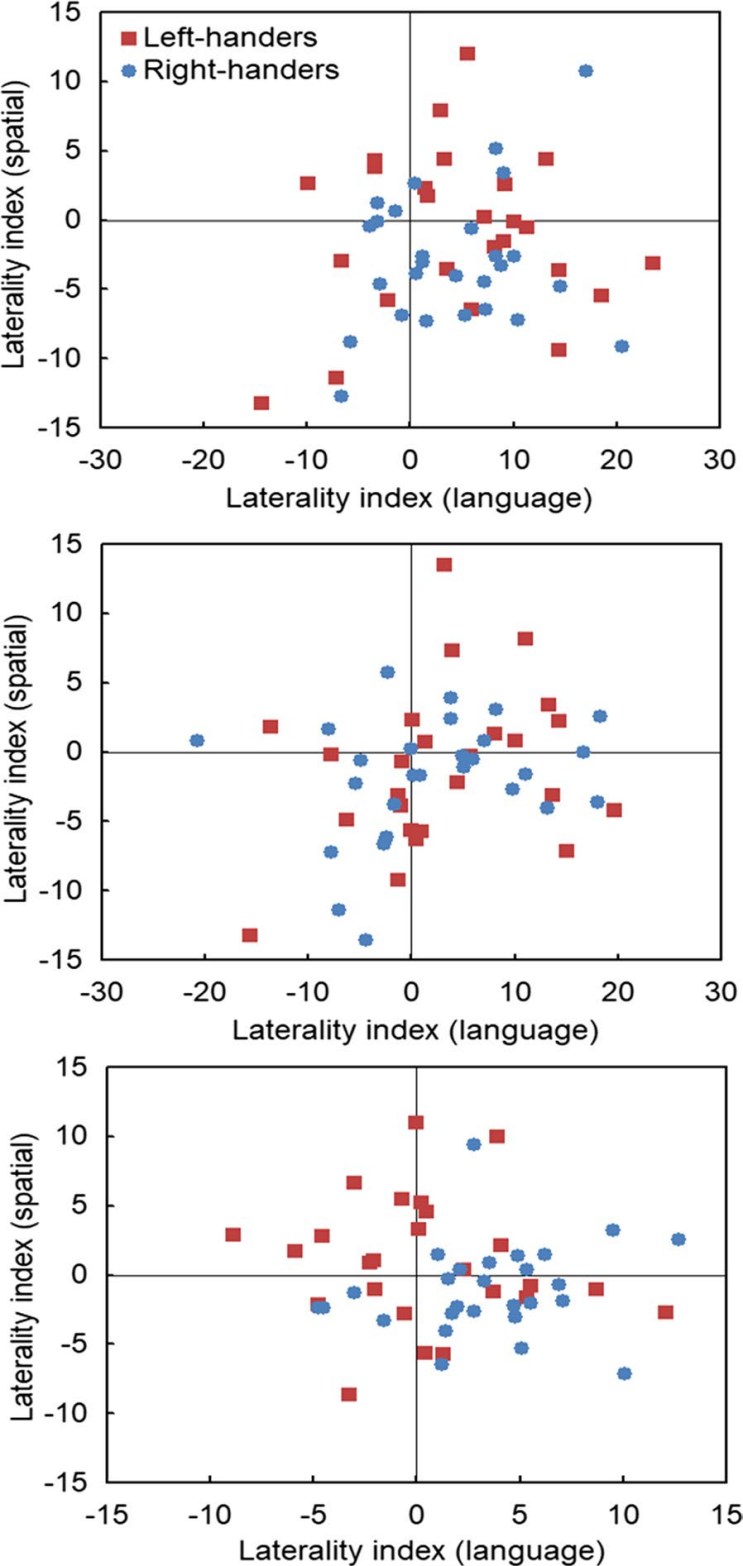
Scatter plots of the laterality indexes for word and symmetric targets alongside incongruent (top panel), congruent (middle panel) and perceptual (lower panel) distractors. The quadrants show the different combinations of complementary lateralisation. Language responses with positive scores represent dominance of the right visual field (left hemisphere) whereas visuospatial responses with negative scores represent dominance of the left visual field (right hemisphere)

Correlation analyses between the LI_TIM_ of the language (word targets) and spatial (symmetric targets) tasks for each distractor condition showed no significant associations, *p* > 0.05. Although many participants and especially right-handers displayed typical complementary lateralisation (i.e., dominance of left hemisphere for language task vs. right hemisphere for visuospatial task), the other three relationships were also noted, i.e., crowding in the left or right hemisphere, and mirror-reversal of both functions. Scatter plots for the different distractor conditions are shown in Fig. 5.

#### Individual- and population-level analysis

1. *Incongruent distractors* (Fig. 5, top panel). First, at the individual level, typical organisation was more prominent for right- than left-handers. That is, although (a)typical complementarity was present as opposed to non-lateralisation (*N* = 3), there were differences between left- and right-handers. In particular, typical complementary lateralisation was more observed for right-handers (*N* = 15, 56%) than left-handers (*N* = 9, 36%), *χ*^2^ = 8.05, *p* < 0.005. In addition, left-hemispheric crowding was more noticed for left-handers (*N* = 7, 28%) than right-handers (*N* = 4, 15%), *χ*^2^ = 5.01, *p* < 0.03. Second, at the population level, many participants showed typical organisation (*N* = 24, 46%) against the null-hypothesis with 20% according to five categories, *z* = 4.69, *p* < 0.0001, 95% CI: 32.09–60.38.
2. *Congruent distractors* (Fig. 5, middle panel). First, at the individual-level, typical organisation was comparable for left- and right-handers. That is, although (a)typical complementarity was present as opposed to non-lateralisation (*N* = 6), there were no differences between left- and right-handers. In particular, typical complementary lateralisation was equally observed for right-handers (*N* = 8, 30%) and left-handers (*N* = 7, 28%), *p* > 0.05. Second, at the population level, there was no prevalence of typical organisation (*N* = 15, 29%) against the null-hypothesis with 20% according to five categories,* p* > 0.05.
3. *Perceptual distractors* (Fig. 5, lower panel). First, at the individual-level, typical organisation was more expressed by right- than left-handers. That is, although (a)typical complementarity dominated as opposed to non-lateralisation (*N* = 3), there were differences between left- and right-handers. Namely, typical complementary lateralisation was more prominent for right-handers (*N* = 14, 52%) than left-handers (*N* = 7, 28%). *χ*^2^ = 12.00, *p* = 0.0005. In addition, mirror-reversal was present for left-handers only (*N* = 7, 28%) whereas left-hemispheric crowding was observed for right-handers (*N* = 9, 33%) as compared to left-handers (*N* = 4, 16%). *χ*^2^ = 7.81, *p* = 0.005. Second, at the population-level, there was prevalence of typical organisation (*N* = 21, 40%) against the null-hypothesis with 20% according to five categories, *z* = 3.61, *p* < 0.0003, 95% CI: 26.66–54.52.

## Discussion

Hemispheric lateralisation represents an essential principle of brain organisation, providing advantages at the individual-level by increasing brain efficiency and at the population-level by facilitating social coordination (Corballis, 1989; Ghirlanda & Vallortigara, 2004). In the present work, we studied language and visuospatial functions that typically lateralise in opposite hemispheres; a dichotomy that likely played an important role in the evolution towards behavioural specialisation (Hugdahl, 2000). In addition, we tested left- and right-handers in various environmental contexts as we argued that both groups rely on different mechanisms of information processing, which has implications for our understanding of how cognitive lateralisation and handedness evolved.

### Language and visuopatial processing

Language functions are usually more efficiently processed in the left than right hemisphere as evidenced from brain imaging and clinical research (Knecht et al, 2000; Price, 2000; Springer et al., 1999). In particular, research has shown that language production and semantic processing are controlled within anterior regions whereas language comprehension is processed within posterior temporo-parietal areas. Along a similar line, behavioural findings have shown that language-related stimuli are more easily processed when presented in the right than left visual field (Van der Haegen & Brysbaert, 2018). We asked participants to perform a lexical decision-making task that required the identification of words versus non-words. The reaction time data revealed that word targets were faster when presented in the right than left visual field, and quicker than non-word targets except when presented in the left visual field alongside incongruent distractors. This indicates that word processing in the non-dominant hemisphere was disrupted by non-word distractors in the opposite field. In contrast, non-word processing was unaffected by the type of distractor and visual field of presentation. Various theories exist that explain differences between word and non-word recognition; some propose a dual-route of lexical access whereas others advocate separate word and non-word detection mechanisms (Weems & Reggia, 2004). Our findings suggest that both lexical categories are processed differently, with words likely relying more on interhemispheric communication than non-words (Iacoboni & Zaidel, 1996; Mohr et al., 1994). The LI_TIM_ further detailed that the type of distractor influenced the advantage of the right visual field for the processing of word targets, with the strongest effect obtained in the presence of non-word distractors.

Visuospatial functions are often lateralised to the right hemisphere and captured by a left visual field advantage such as for the judgement of line orientation and pre-bisected lines (Atkinson & Egeth, 1973). This bias is proposed to reflect right-hemispheric dominance for visuospatial attention due to asymmetries of the cerebral hemispheres or network connectivity patterns when attending to and processing stimuli in surrounding space (Corbetta & Shulman, 2011; Petersen & Posner, 2012). We tested participants on their ability to detect vertical symmetry; a function that includes identification of objects and their relations in space. Previous work has established dominance of the left visual field for symmetry detection in the majority of the population (Verma et al., 2013). We observed that the reaction time of symmetry detection differed for left- and right-handers as a function of visual field presentation. Whereas right-handers were faster in responding to targets in the left than right visual field, the reaction times of left-handers did not differ between both sides. These differences were supported by the LI_TIM_ data with right-handers benefitting from left visual field dominance as compared to left-handers who lacked a clear hemispheric differentiation. This is in line with research that has revealed that left- and right-handers show dissimilar modulations in alertness (Bareham et al., 2015), which could be due to distinct neural circuits that direct attention to external stimuli (Liu et al., 2009). Further evidence comes from clinical observations demonstrating that handedness impacts the visuospatial abilities of patients with left- and right-hemispheric lesions (Hécaen & Sauguet, 1971).

However, the data from the symmetry task also showed reaction time modulations that were independent of handedness, pointing to a stronger sensitivity of the right than left hemisphere for visuospatial functions (Liu et al., 2009; Prete et al., 2020). In particular, responding to targets in the left versus right visual field varied with distractor type. That is, there were faster responses to left than right visual field targets with incongruent and congruent distractors but not with perceptual distractors, underlining that the characteristics of the stimuli influenced processing. Furthermore, responses to symmetric as opposed to asymmetric targets were faster with congruent distractors whereas no differences were noted alongside perceptual and incongruent distractors. This finding points to the intrinsic and basic nature of symmetry detection (Verma et al., 2013; Wagemans, 1995). It has been proposed that a preference for symmetric configurations occurs due to perceptual dominance for tuning to regularity, which guides figure-ground segmentation (Kootstra et al., 2011) and the organisation of the principal axis of shape (Marr, 1982).

### Lateralisation of language and visuospatial processing

There were common findings across language and visuospatial functions, suggesting that moderators play a guiding role in how hemispheric resources are used. A first finding represented the involvement of dynamic interhemispheric interactions. In particular, target processing was shaped by the presence of the distractor in the opposite field, capturing between-hemispheric communication**.** Furthermore, hemispheric asymmetry was strongest when the distractor was category-related to the target (non-perceptual). This observation underlines that interhemispheric exchanges of stimulus attributes are differential and adaptive, supporting a unified experience from the environment (Bergert, 2010).

A second finding was that the non-dominant hemisphere was more affected by the distractors than the dominant hemisphere (Chiarello & Maxfield, 1996). This suggests an asymmetric sensitivity of functional differences between hemispheres and may relate to the vulnerability of the non-dominant hemisphere in two ways: (a) interference during transfer of information to the dominant hemisphere, or, (b) obstruction of transfer that forces the non-dominant hemisphere to target processing (Iacoboni & Zaidel, 1996). This would provide support to theories of inhibition, proposing that the dominant hemisphere triggers an inhibitory influence for minimising negative effects from the non-dominant hemisphere (Weems & Reggia, 2004).

A third finding was that individual-level lateralisation (left or right) was present as compared to non-lateralisation, albeit with right-handers showing superiority of the dominant hemisphere whereas left-handers manifested equal preference for either side. However, the data at the population-level underlined convergence towards the same bias across individuals, confirming the dominant hemisphere as specialised for the information processing. Theoretical models propose that the directional alignment at the population-level may have evolved due to social pressures that force individuals to adapt to one another and align their behaviour (Ghirlanda & Vallortigara, 2004). Therefore, once an individual-level preference is adopted at the population-level, there would be no further need for alignment as any interactions would be integrated into the existing organisation (Frasnelli, 2013). Thus, a population consisting of left- and right-lateralised individuals in unequal numbers can be evolutionarily stable on the basis of strategic factors that arise from frequency-dependent costs and benefits (Ghirlanda & Vallortigara, 2004).

### Complementary language and visuospatial processing

We observed that (a)typical relationships of both cognitive functions varied as a function of the target–distractor combination alongside handedness. That the complementary organisation relied on distractor type confirms a dynamic role of interhemispheric interactions in defining lateralisation properties (Tzourio-Mazoyer, 2016), and could represent a potential marker of functional lateralisation. This premise is supported in our study by the data from the congruent condition in which identical stimuli were presented to both visual fields, providing redundant information. In this case, both left- and right-handers showed a mixed range of individual patterns with a lack of convergence towards a typical profile at the population-level. In contrast, for the incongruent and perceptual conditions, which included distinct stimuli to both visual fields, typical complementary lateralisation was more present for right- than left-handed individuals, with the dominant profile confirmed at the population-level. Some atypical patterns were more observed than others, i.e., crowding in the left hemisphere (left- and right-handers) and mirror-reversal (left-handers). These atypical clusters indicate that, although typical complementary lateralisation is generally a stable feature across individuals, flexibility exists that is more prominent for left- than right-handers. Thus, non-right-handedness is characterised by a higher degree of individual variation as well as deviation from a typical organisation for these cognitive functions.

To explain complementarity, two main viewpoints exist: statistical and causal influences. Whereas, the statistical hypothesis proposes that lateralisation of different functions take their own course (Bryden et al., 1983; Groen et al., 2012; Levy, 1969; Teuber, 1974)**,** the causal hypothesis suggests that functions do not lateralise independently (Cai et al., 2013). Our results did not show significant relationships between the LI_TIM_ of both functions, suggesting that neither hypothesis can fully account for the data. In particular, these findings are in line with suggestions that hemispheric asymmetries interact but rely on multiple influences (Badzakova-Trajkov et al., 2010; Gerrits et al., 2020). However, we acknowledge that a larger sample size would be required in order to confirm reliable population estimates of each pattern’s prevalence.

### Handedness and its links with language and visuospatial processing

Handedness is one of the strongest human behavioural asymmetries (Triggs et al., 2000) and is considered to either follow from or cause asymmetries in the organisation of the brain, with reciprocal influences between both levels (Andersen & Siebner, 2018; Vingerhoets, 2019). Handedness is usually quantified with respect to the preference of one hand to perform manual tasks, or, the performance that characterises the hand that is most skilled at doing those tasks (Corey et al., 2001). Hand preference and performance are thus related but also reflect distinctive entities of handedness, and may vary at the individual level.

The handedness tests showed a positive correlation between the questionnaire and pegboard data, with increased right-handedness associating with a stronger right hand performance for the pegboard task. These findings indicate that hand preference linked with asymmetries of hand performance, but the agreement is not perfect and shows unshared variance (Annett, 2002). Noteworthy is that a distinctive number of left-handers performed as right-handers and obtained a right hand advantage, which reflects the existence of a range of lateralised performances. In our study, these participants involved consistent as well as inconsistent left-handers, suggesting that the performance shift was not due to hand use per se. Therefore, the results underline that handedness represents a multifaceted concept.

There were specific links between handedness and the cognitive functions**.** *First,* many right-handers demonstrated dominance of the right visual field for language processing and left visual field for visuospatial processing whereas left-handers showed bimodal frequency distributions, indicating left- and right-type individuals. *Second*, the handedness-language data revealed positive associations between dominance of the right visual field for word processing with a right hand preference (questionnaire), and a right hand performance advantage (pegboard). These results denote a relationship between left-hemispheric dominance for language (at least for manual action verbs) and both right-handedness and right hand dexterity. This link could be due to a common basis, for example through a system that governs manual gestures and language (Corballis et al., 2012). In particular, both activities are controlled by shared circuitry in left-lateralised areas (Xu et al., 2009) and handedness for gestures and hemispheric dominance for language are related (Kimura, 1973a, 1973b). In addition, for the handedness-visuospatial data, a negative association was noted between dominance of the left visual field for symmetry detection with a right hand preference from the questionnaire (i.e., hand selection), but not with right hand dexterity from the pegboard task (i.e., hand performance). This is in line with work that suggests a link between hand preference and selective fronto-parietal pathways involved in attentional processing of goal-directed movements (Howells et al., 2018, 2020).

To clarify these results, it is argued that early biases of visual attention modulate the development of motor asymmetries towards the right hand later in life, with right-handedness arising from a rightward attentional preference (Marzoli et al., 2014). This shared process could also be interpreted as joint attention for mutual understanding during interactive activities (Gong & Shuai, 2012). Together, our data propose a link between the language system with right hand use alongside the attention system that guides our hand-space patterns. To support neural coupling between the language and attention systems, interface regions such as the visual word form area could play a significant role in providing a computational node that couples language and attention circuits (Chen et al., 2019).

Thus, the data argue for intricate dependences between manual, language and attention systems. It is likely that these share a common evolutionary origin, with right hand use during communicative activities alongside attentional preferences toward space that support those settings. Overall, the present findings contribute to our understanding of the natural expression of hemispheric asymmetries and the processes by which handedness and other cognitive functions are coupled with one another. These mechanisms bias information processing and accordingly influence our choices, strategies and behaviours in everyday activities.

In conclusion, it is acknowledged that cognitive functions such as language and visuospatial attention are usually asymmetrically organised in the brain. We observed that lateralisation of these cognitive functions as well as their relationship followed a left–right organisation, but with variation such that a portion of the participants showed distinct processing. In particular, right-handed individuals generally tended towards typical profiles whereas left-handers demonstrated increased individual variation and atypical organisation. That atypical variants differed with the target–distractor combination underlines a key role of interhemispheric communication in characterising lateralisation properties. Therefore, both intrahemispheric and interhemispheric mechanisms are critically involved in defining lateralised cognition. The results further revealed distinctive relationships between right-handedness and left-hemispheric dominance for language alongside right-hemispheric dominance for visuospatial processing. Together, these findings illustrate the role of broader mechanisms in supporting hemispheric lateralisation of cognition and behaviour, relying on common principles but controlled by internal and external factors, which accordingly steer our decisions, preferences and experiences in day-to-day settings.

## Supplementary Information

**Additional file 1.** Accuracy data of the language and visuospatial tasks.

## Data Availability

The data are stored on the Open Source Framework (OSF): https://osf.io/gecfx/.

## References

[CR1] Andersen KW, Siebner HR (2018). Mapping dexterity and handedness: Recent insights and future challenges. Current Opinion in Behavioral Sciences.

[CR2] Annett M (2002). Handedness and brain asymmetry: The right shift theory.

[CR3] Atkinson J, Egeth H (1973). Right hemisphere superiority in visual orientation matching. Canadian Journal of Psychology.

[CR4] Badzakova-Trajkov G, Häberling IS, Roberts RP, Corballis MC (2010). Cerebral asymmetries: Complementary and independent processes. PLoS ONE.

[CR5] Bareham CA, Bekinschtein TA, Scott SK, Manly T (2015). Does left-handedness confer resistance to spatial bias?. Scientific Reports.

[CR6] Bergert S (2010). Do our brain hemispheres exchange some stimulus aspects better than others?. Neuropsychologia.

[CR7] Bloom JS, Hynd GW (2005). The role of the corpus callosum in interhemispheric transfer of information: Excitation or inhibition?. Neuropsychology Review.

[CR8] Boles, D. B. (1990). What bilateral displays do. *Brain and Cognition, 12*, 205–228. 10.1016/0278-2626(90)90016-h. 10.1016/0278-2626(90)90016-h 2340152

[CR9] Bryden MP (1982). Laterality: Functional asymmetry in the intact brain.

[CR10] Bryden MP, Hecaen H, DeAgostini M (1983). Patterns of cerebral organization. Brain and Language.

[CR11] Cai Q, Van der Haegen L, Brysbaert M (2013). Complementary hemispheric specialization for language production and visuospatial attention. Proceedings of the National Academy of Sciences USA.

[CR12] Chen L, Wassermann D, Abrams DA, Kochalka J, Gallardo-Diez G, Menon V (2019). The visual word form area (VWFA) is part of both language and attention circuitry. Nature CommunIcations.

[CR13] Cherbuin N, Brinkman C (2006). Hemispheric interactions are different in left-handed individuals. Neuropsychology.

[CR14] Chiarello C, Maxfield L (1996). Varieties of interhemispheric inhibition, or how to keep a good hemisphere down. Brain and Cognition.

[CR15] Clarke JM, Zaidel E (1994). Anatomical–behavioral relationships: Corpus callosum morphometry and hemispheric specialization. Behavioural Brain Research.

[CR16] Corballis MC (1989). Laterality and human evolution. Psychological Reviews.

[CR17] Corballis MC, Badzakova-Trajkov G, Häberling IS (2012). Right hand, left brain: Genetic and evolutionary bases of cerebral asymmetries for language and manual action. Wiley Interdisciplinary Reviews: Cognitive Sciences.

[CR18] Corbetta M, Shulman GL (2011). Spatial neglect and attention networks. Annual Review of Neuroscience.

[CR19] Coren, S., & Porac, C. (1997). Fifty centuries of right-handedness: The historical record. Science, 198, 631–632. 10.1126/science.335510. 10.1126/science.335510 335510

[CR20] Corey, D. M., Hurley, M. M., & Foundas, A. L. (2001). Right and left handedness defined: a multivariate approach using hand preference and hand performance measures. *Neuropsychiatry, Neuropsychology and Behavioral Neurology, 14*, 144–152. 11513097

[CR21] Flöel A, Jansen A, Deppe M, Kanowski M, Konrad C, Sommer J, Knecht S (2005). Atypical hemispheric dominance for attention: Functional MRI topography. Journal of Cerebral Blood Flow & Metabolism.

[CR22] Frasnelli E (2013). Brain and behavior in invertebrates. Frontiers in Psychology.

[CR23] Gerrits R, Verhelst H, Vingerhoets G (2020). Mirrored brain organization: Statistical anomaly or reversal of hemispheric functional segregation bias?. Proceedings of the National Academy of Sciences USA.

[CR24] Ghirlanda S, Vallortigara G (2004). The evolution of brain lateralization: A game-theoretical analysis of population structure. Proceedings of the Biological Sciences.

[CR25] Gong T, Shuai L (2012). Modelling the coevolution of joint attention and language. Proceedings of the Royal Society B.

[CR26] Groen MA, Whitehouse AJ, Badcock NA, Bishop DV (2012). Does cerebral lateralization develop? A study using functional transcranial Doppler ultrasound assessing lateralization for language production and visuospatial memory. Brain and Behavior.

[CR27] Hauk O, Johnsrude I, Pulvermüller F (2004). Somatotopic representation of action words in human motor and premotor cortex. Neuron.

[CR28] Hécaen H, Sauguet J (1971). Cerebral dominance in left-handed subjects. Cortex.

[CR29] Howells H, Puglisi G, Leonetti A, Vigano L, Fornia L, Simone L, Forkel SJ, Rossi M, Riva M, Cerri G, Bello L (2020). The role of left fronto-parietal tracts in hand selection: Evidence from neurosurgery. Cortex.

[CR30] Howells H, Thiebaut de Schotten M, Dell'Acqua F, Beyh A, Zappalà G, Leslie A, Simmons A, Murphy DG, Catani M (2018). Frontoparietal tracts linked to lateralized hand preference and manual specialization. Cerebral Cortex.

[CR31] Hugdahl K (2000). Lateralization of cognitive processes in the brain. Acta Psychologica.

[CR32] Hunter ZR, Brysbaert M (2008). Visual half-field experiments are a good measure of cerebral language dominance if used properly: Evidence from fMRI. Neuropsychologia.

[CR33] Iacoboni M, Zaidel E (1996). Hemispheric independence in word recognition: Evidence from unilateral and bilateral presentations. Brain and Language.

[CR34] Jäncke L, Peters M, Schalug G, Posse S, Steinmetz H, Müller-Gärtner HW (1998). Differential magnetic resonance signal change in human sensorimotor cortex to finger movements of different rate of the dominant and subdominant hand. Cognitive Brain Research.

[CR35] Karolis VR, Corbetta M, Thiebout de Schotten M (2019). The architecture of functional lateralisation and its relationship to callosal connectivity in the human brain. Nature Communications.

[CR36] Kimura D (1973). Manual activity during speaking. I. Right-handers. Neuropsychologia.

[CR37] Kimura D (1973). Manual activity during speaking. II. Left-handers. Neuropsychologia.

[CR38] Kinsbourne M, Jeannerod M (1987). Mechanisms of unilateral neglect. Neurophysiological and neuropsychological aspects of spatial neglect.

[CR39] Knecht S, Dräger B, Deppe M, Bobe L, Lohmann H, Flöel A, Ringelstein E, Henningsen H (2000). Handedness and hemispheric language dominance in healthy humans. Brain.

[CR40] Kootstra G, de Boer B, Schomaker LRB (2011). Predicting eye fixations on complex visual stimuli using local symmetry. Cognitive Computation.

[CR41] Kosslyn SM (1987). Seeing and imagining in the cerebral hemispheres: A computational approach. Psychological Review.

[CR42] Levy J (1969). Possible basis for the evolution of lateral specialization of the human brain. Nature.

[CR43] Liu H, Stufflebeam SM, Sepulcre J, Hedden T, Buckner RL (2009). Evidence from intrinsic activity that asymmetry of the human brain is controlled by multiple factors. Proceedings of the National Academy of Sciences USA.

[CR44] Marr D (1982). Vision: A computational investigation into the human representation and processing of visual information.

[CR45] Martin K, Jacobs S, Frey SH (2011). Handedness-dependent and -independent cerebral asymmetries in the anterior intraparietal sulcus and ventral premotor cortex during grasp planning. NeuroImage.

[CR46] Marzoli D, Prete G, Tommasi L (2014). Perceptual asymmetries and handedness: A neglected link?. Frontiers in Psychology.

[CR47] Measso G, Zaidel E (1990). Effect of response programming on hemispheric differences in lexical decision. Neuropsychologia.

[CR48] Michel, G. F. (2021). Handedness development: A model for investigating the development of hemispheric specialization and interhemispheric coordination. *Symmetry.* https://www.mdpi.com/2073-8994/13/6/992

[CR49] Mohr B, Pulvermüller F, Zaidel E (1994). Lexical decision after left, right, and bilateral presentation of function words, content words and non-words: Evidence for interhemispheric interaction. Neuropsychologia.

[CR50] O’Regan L, Serrien DJ (2018). Individual differences and hemispheric asymmetries for language and spatial attention. Frontiers in Human Neuroscience.

[CR51] Peirce JW, MacAskill MR (2018). Building experiments in PsychoPy.

[CR52] Perrone-Bertolotti M, Lemonnier S, Baciu M (2013). Behavioral evidence for inter-hemispheric cooperation during a lexical decision task: A divided visual field experiment. Frontiers in Human Neuroscience.

[CR53] Petersen SE, Posner MI (2012). The attention system of the human brain: 20 years after. Annual Review of Neuroscience.

[CR54] Pool EM, Rehme AK, Fink GR, Eickhoff SB, Grefkes C (2014). Handedness and effective connectivity of the motor system. NeuroImage.

[CR55] Powell JL, Kamp GJ, García-Finaña M (2012). Association between language and spatial laterality and cognitive ability: An fMRI study. NeuroImage.

[CR56] Prete, G., Fabri, M., & Tommasi, L. (2020). Asymmetry for symmetry: Right-hemispheric superiority in bi-dimensional symmetry perception. *Symmetry*. https://www.mdpi.com/2073-8994/9/5/76

[CR57] Price CJ (2000). The anatomy of language: Contributions from functional neuroimaging. The Journal of Anatomy.

[CR58] Serrien DJ, Sovijärvi-Spapé MM (2016). Manual dexterity: Functional lateralisation patterns and motor efficiency. Brain and Cognition.

[CR59] Springer JA, Binder JR, Hammeke TA, Swanson SJ, Frost JA, Bellgowan PS, Brewer CC, Perry HM, Morris GL, Mueller WM (1999). Language dominance in neurologically normal and epilepsy subjects: A functional MRI study. Brain.

[CR60] Teuber HL, Schmidts FO, Worden FG (1974). Why two brains?. The neurosciences: Third study program.

[CR61] Thiebaut de Schotten M, Dell'Acqua F, Forkel SJ, Simmons A, Vergani F, Murphy DGM, Catani M (2011). A lateralized brain network for visuospatial attention. Nature Neuroscience.

[CR62] Triggs W, Calvanio R, Levine M, Heaton R, Heilman K (2000). Predicting hand preference with performance on motor tasks. Cortex.

[CR63] Tussis L, Sollmann N, Boeckh-Behrens T, Meyer B, Krieg SM (2016). Language function distribution in left-handers: A navigated transcranial magnetic stimulation study. Neuropsychologia.

[CR64] Tzourio N, Crivello F, Mellet E, Nkanga-Ngila B, Mazoyer B (1998). Functional anatomy of dominance for speech comprehension in left handers versus right handers. NeuroImage.

[CR65] Tzourio-Mazoyer N, Kennedy H, Van Essen DC, Christen Y (2016). Intra- and inter-hemispheric connectivity supporting hemispheric specialization. Micro-, meso- and macro-connectomics of the brain. Research and perspectives in neurosciences.

[CR66] Van der Haegen L, Brysbaert M (2018). The relationship between behavioral language laterality, face laterality and language performance in left-handers. PLoS ONE.

[CR67] Verma A, Van der Haegen L, Brysbaert M (2013). Symmetry detection in typically and atypically speech lateralized individuals: A visual half-field study. Neuropsychologia.

[CR68] Vingerhoets G (2019). Phenotypes in hemispheric functional segregation? Perspectives and challenges. Physics of Life Reviews.

[CR69] Vogel JJ, Bowers CA, Vogel DS (2003). Cerebral lateralization of spatial abilities: A meta-analysis. Brain and Cognition.

[CR70] Wagemans J (1995). Detection of visual symmetries. Spatial Vision.

[CR71] Weems SA, Reggia JA (2004). Hemispheric specialization and independence for word recognition: A comparison of three computational models. Brain and Language.

[CR72] Wilkinson DT, Halligan PW (2002). The effects of stimulus symmetry on landmark judgments in left and right visual fields. Neuropsychologia.

[CR73] Xu J, Patrick J, Gannon PJ, Emmorey K, Smith JF, Allen R, Braun AR (2009). Symbolic gestures and spoken language are processed by a common neural system. Proceedings of the National Academy of Sciences USA.

