# Supplementary material for: The interactive functional biases of manual, language and attention systems
Source: Cogn Res Princ Implic. 2022 Mar 2;7:20. doi: 10.1186/s41235-022-00365-x (PMC8891409; doi:10.1186/s41235-022-00365-x)
Supplement: Supplementary file 1 — Additional file 1. Accuracy data of the language and visuospatial tasks. [file 41235_2022_365_MOESM1_ESM.doc]

**Supplementary material**

**Results**

**Language task: Accuracy**

*Mean accuracy*. There were significant main effects of Visual Field, *F*(1,50)=8.85, *p*=.004, *η^2^p*= 0.15, Target, *F*(1,50)=10.67, *p*=0.002, *η^2^p*=0.18, Distractor, *F*(2,100)=10.18, p<0.001, *η^2^p*=0.17, and interactions of Visual Field x Target, *F*(1,50)=11.08, *p*=0.002, *η^2^p*=0.18, and Target x Distractor, *F*(2,100)=11.63, p<0.001, *η^2^p*=0.18. There was also a significant Visual Field x Target x Distractor interaction, *F*(2,100)=8.90, *p*<0.001, *η^2^p*=0.19 (Fig. 1), showing that responses to word targets were most accurate in the right visual field whereas those to non-word targets had similar accuracy across visual fields. In addition, word targets were more accurate than non-word targets, except in the left visual field alongside incongruent (p<0.001) and congruent (*p*=0.003) distractors. The mean accuracy scores in the left and right visual field for non-words were 82±1% and 77±1% (incongruent), 79±2% and 77±2% (congruent), 76±1% and 82±2% (perceptual). For words the scores were 77±2% and 85±1% (incongruent); 75±2% and 82±2% (congruent); 85±1% and 90±1% (perceptual).





Fig. 1. Accuracy scores for word and non-word targets as a function of visual field of presentation for incongruent (Inc), congruent (Con) and perceptual (Per) conditions.

*LI_ACC_ (word targets), LI_ACC_ (word targets) and handedness*. No effects were observed, *p*>0.05.

**Visuospatial task: Accuracy**

*Mean accuracy*. There was a significant main effect of Distractor, *F*(2,100)=3.67, *p*=0.28, *η^2^p*=0.07 as well as significant interactions of Visual Field x Distractor, *F*(2,100)=3.61, *p*=0.03, *η^2^p*=0.07, and Target x Distractor, *F*(2,100)=8.55, p<0.001, *η^2^p*=0.15. There was also a significant Visual Field x Target x Distractor interaction, *F*(2,100)=16.76, *p*<0.01, *η^2^p*=0.25, indicating that left visual field responses were similar for symmetric targets (incongruent: 85±2%; congruent: 87±1%; perceptual: 85±2%) and asymmetric targets (incongruent: 86±1%; congruent: 86±1%; perceptual: 89±1%). In the right visual field, responses to symmetric vs. asymmetric targets were less accurate for incongruent condition (79±2% vs. 87±1%) whereas responses to asymmetric vs. symmetric targets were less accurate for perceptual condition (79±2% vs. 88±1%, *p*<0.05), and no difference for congruent condition (asymmetric: 87±1%; symmetric: 87±2%).

*LI_ACC_ (symmetric targets).* There was a significant main effect of Distractor, *F*(2,100)=5.90, *p*=0.004, *η^2^p*=0.11, showing a left-to-right shift of dominance as a function of type of distractor (incongruent: -3.0±1.2; congruent: -0.1±0.9; perceptual: 1.8±0.9) with a difference between the former and latter condition, *p<*0.003.

*LI_ACC_ (symmetric targets) and handedness*. Correlation analysis between LI_ACC_ and LI*_PEG_* (manual dexterity) and between LI_ACC_ and handedness scores revealed no effect, *p*>0.05.
